# Advancing One Health in Africa through continental early warning environmental surveillance

**DOI:** 10.1128/aem.00471-26

**Published:** 2026-07-01

**Authors:** John Bosco Kalule

**Affiliations:** 1 Department of Biotechnical and Diagnostic Sciences (BDS), College of Veterinary Medicine, Animal Resources and Biosecurity (CoVAB), Makerere University 58588, Kampala, Uganda; Michigan State University, East Lansing, Michigan, USA

**Keywords:** environmental surveillance in Africa

## Abstract

Environmental surveillance is rapidly emerging as one of the most transformative tools for infectious disease detection, especially in Africa. In their minireview published in *Applied and Environmental Microbiology*, E. Darko, D. Akortia, G. Nkrumah, F. Opoku Agyapong, et al. (Appl Environ Microbiol 92:e01932-25, 2026, https://doi.org/10.1128/aem.01932-25) synthesize evidence from 90 studies across Africa and demonstrate both the promise and fragmentation of pathogen-focused environmental surveillance. Their findings reveal major inequities but also underscore a critical opportunity as relates to the development of integrated, multi-pathogen environmental surveillance systems capable of strengthening continent-wide early warning systems.

## COMMENTARY

A recent minireview by Darko and colleagues published in *Applied and Environmental Microbiology* represents a timely and important contribution to the evolving field of environmental microbiology in Africa. By systematically synthesizing evidence from 90 studies spanning 17 African countries, the authors provide one of the most comprehensive overviews to date of pathogen-focused environmental surveillance (ES) activities on the continent (1). Their work arrives at a pivotal moment when wastewater and environmental surveillance have transitioned from niche epidemiological tools into central components of global outbreak preparedness strategies, as was evidenced during the COVID-19 pandemic.

The COVID-19 pandemic fundamentally altered perceptions of environmental surveillance. Wastewater-based epidemiology demonstrated that pathogens could be detected at the population level even before symptomatic clinical cases overwhelmed healthcare systems (2). In many regions, environmental surveillance provided near-real-time insights into viral circulation, transmission dynamics, and variant emergence (3). And yet, as the review clearly demonstrates, Africa’s participation in this surveillance revolution remains uneven (skewed to the more developed nations such as South Africa) and underdeveloped (1).

A major strength of the review lies in its broad pathogen scope. Previous reviews frequently focused on single organisms such as poliovirus or SARS-CoV-2, but this synthesis captures well clinically relevant bacteria, viruses, fungi, and parasites within a unified framework.

The identification of 47 microbial species across diverse environmental matrices illustrates the remarkable breadth of pathogens that can be monitored through ES. Particularly notable among them is the prominence of pathogens with substantial epidemic potential, including SARS-CoV-2, poliovirus, rotavirus, *Vibrio cholerae*, and *Salmonella* Typhi (1).

Equally important are the geographic insights generated by the review. Only 17 out of 54 African countries were represented among eligible studies, with South Africa, Egypt, Ghana, and Kenya accounting for a disproportionate share of publications (1). This concentration of ES capacity reflects broader structural inequalities in laboratory infrastructure, molecular diagnostic access, research financing, and wastewater management systems across the continent (4). It is worth noting with concern that large portions of Central Africa and the Sahel remain essentially absent from the environmental surveillance literature despite experiencing recurrent outbreaks of cholera, typhoid fever, viral hemorrhagic fevers, and antimicrobial-resistant infections.

Importantly, the review also exposes a critical urban bias in African ES activities. More than 80% of studies were conducted in urban settings, while rural environments where sanitation infrastructure is often weakest and zoonotic interfaces are intense remain severely underrepresented (1). This imbalance has important epidemiological implications because rural communities frequently possess reservoirs/ecological niches for neglected tropical diseases, enteric pathogens, and emerging zoonoses; yet they are largely invisible within current environmental monitoring systems. Future surveillance frameworks must therefore move beyond centralized municipal wastewater systems toward decentralized, community-based sampling models capable of functioning in informal settlements, peri-urban communities, specialized settings such as refugee camps, and remote rural ecosystems.

Methodologically, the review highlights both innovation and fragmentation on the African continent. The prevalent use of grab sampling and PCR-based detection methods demonstrates the increasing accessibility of molecular diagnostics across African laboratories. However, the diversity of concentration techniques and laboratory workflows also reveals a lack of harmonization that may hinder cross-country comparisons and regional integration. Standardization remains one of the greatest obstacles to transforming ES from isolated research initiatives into interoperable continental surveillance systems (5, 6).

In this study, the inclusion of studies identifying antimicrobial resistance (AMR) genes linked to specific pathogens is particularly significant (1). Although studies that focused exclusively on AMR genes were excluded, the review recognizes the growing intersection between pathogen surveillance and antimicrobial resistance monitoring. This distinction is important because wastewater environments increasingly function as reservoirs and mixing grounds for resistant micro-organisms and other AMR determinants such as heavy metals (7). Environmental surveillance therefore offers a unique opportunity to integrate infectious disease monitoring with AMR surveillance under a unified One Health framework.

Arguably, the most important implication of this review is its demonstration that environmental surveillance in Africa is already operational, but very fragmented across the continent. The major challenge ahead is not whether ES can work in African settings, but how it can be scaled sustainably and equitably for wider deployment across the continent to permit continent-wide co-benefits. For example, the review provides compelling evidence that ES can support early outbreak detection, monitor pathogen circulation, evaluate intervention effectiveness, and complement Integrated Disease Surveillance and Response (IDSR) systems (1).

Noteworthy, sustainability remains uncertain because much of the continent’s ES expansion during the COVID-19 era was driven by emergency funding and externally supported initiatives with limited local contribution. Without long-term institutionalization and localized funding strategies, many of these surveillance systems risk collapse.

On this note, African governments and regional public health institutions such as Africa CDC and PulseNet Africa must therefore transition ES from short-term research projects into permanent continent- or region-wide public health infrastructure (8).

**Fig 1:**
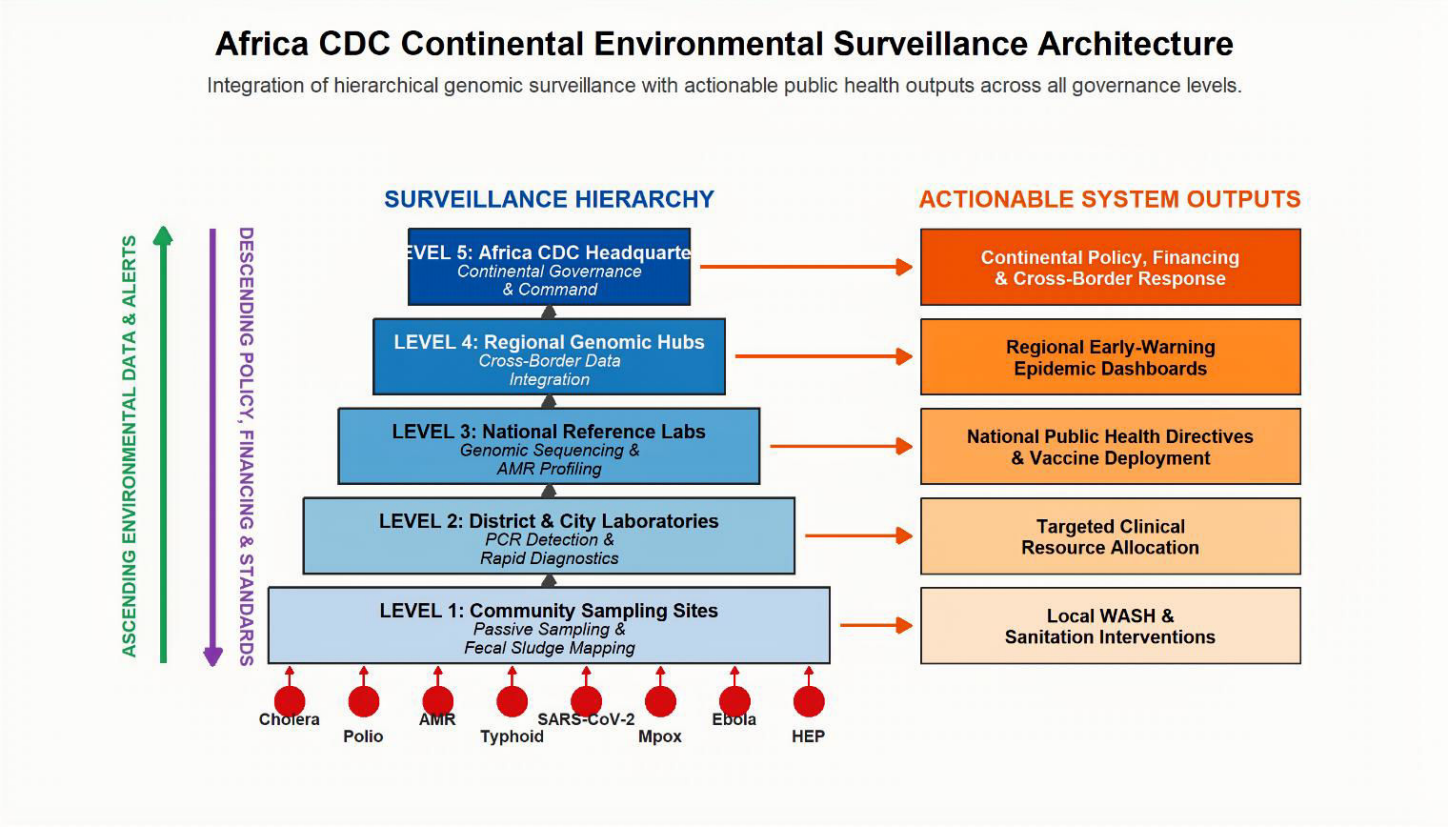
Africa continental environmental surveillance architecture.

To promote wastewater and environmental surveillance in Africa, in 2025, the Africa CDC developed a roadmap to map environmental and wastewater surveillance capacity, identify gaps in infrastructure, and establish harmonized technical guidance across African Union member states. The strategy focuses on building sustainable financing, creating monitoring frameworks, and integrating these systems directly into existing national Integrated Disease Surveillance and Response (IDSR) structures. Crucially, the Africa CDC emphasized that this roadmap must be context-specific and adapted to African realities rather than simply replicating high-income country models. To address the continent’s diverse sanitation landscape, the roadmap promotes flexible, locally adaptable surveillance models capable of functioning across informal settlements, decentralized networks, and rural environments (Fig. 1).

Importantly, the next generation of African ES systems should also embrace ecological and climatic intelligence because increasing urbanization, flooding, population displacement, and climate-driven sanitation disruptions are reshaping pathogen transmission dynamics across the continent (9). Environmental surveillance is uniquely positioned to capture these changing ecological warning signs because it measures population-level pathogen circulation directly within their environmental reservoirs.

The rapid review by Darko and colleagues provides a crucial blueprint for the future of infectious disease surveillance in Africa, highlighting a unique opportunity to build adaptive, decentralized, and One Health-oriented workflows that are tailored specifically to the African continent’s epidemiological realities rather than merely replicating developed nation models. To fully realize this potential, the critical task moving forward is to strategically channel the momentum documented in this review into synchronized, locally adapted, durable, integrated continental workflows, transforming what has historically been a small cluster of isolated success stories into a continent-wide early warning system managed under the Africa CDC.
